# Molecular Studies and an *ex vivo* Complement Assay on Endothelium Highlight the Genetic Complexity of Atypical Hemolytic Uremic Syndrome: The Case of a Pedigree With a Null CD46 Variant

**DOI:** 10.3389/fmed.2020.579418

**Published:** 2020-11-03

**Authors:** Rossella Piras, Paraskevas Iatropoulos, Elena Bresin, Marta Todeschini, Sara Gastoldi, Elisabetta Valoti, Marta Alberti, Caterina Mele, Miriam Galbusera, Paola Cuccarolo, Ariela Benigni, Giuseppe Remuzzi, Marina Noris

**Affiliations:** Clinical Research Center for Rare Diseases ‘Aldo e Cele Daccò,’ Istituto di Ricerche Farmacologiche Mario Negri IRCCS, Bergamo, Italy

**Keywords:** atypical hemolytic uremic syndrome, complement, membrane cofactor protein, incomplete penetrance, splicing, CD46 expression, *ex-vivo* assay, rare variants

## Abstract

Atypical hemolytic uremic syndrome (aHUS) is an ultra-rare disease characterized by microangiopathic hemolysis, thrombocytopenia, and renal impairment and is associated with dysregulation of the alternative complement pathway on the microvascular endothelium. Outcomes have improved greatly with pharmacologic complement C5 blockade. Abnormalities in complement genes (*CFH, CD46, CFI, CFB, C3*, and *THBD*), *CFH–CFHR* genomic rearrangements, and anti-FH antibodies have been reported in 40–60% of cases. The penetrance of aHUS is incomplete in carriers of complement gene abnormalities; and multiple hits, including the *CFH–H3* and *CD46*_*GGAAC*_ risk haplotypes and the CFHR1^*^B risk allele, as well as environmental factors, contribute to disease development. Here, we investigated the determinants of penetrance of aHUS associated with *CD46* genetic abnormalities. We studied 485 aHUS patients and found *CD46* rare variants (RVs) in about 10%. The c.286+2T>G RV was the most prevalent (13/485) and was associated with <30% penetrance. We conducted an in-depth study of a large pedigree including a proband who is heterozygous for the c.286+2T>G RV who experienced a severe form of aHUS and developed end-stage renal failure. The father and paternal uncle with the same variant in homozygosity and six heterozygous relatives are unaffected. Flow cytometry analysis showed about 50% reduction of CD46 expression on blood mononuclear cells from the heterozygous proband and over 90% reduction in cells from the proband's unaffected homozygous father and aunt. Further genetic studies did not reveal RVs in known aHUS-associated genes or common genetic modifiers that segregated with the disease. Importantly, a specific *ex vivo* test showed excessive complement deposition on endothelial cells exposed to sera from the proband, and also from his mother and maternal uncle, who do not carry the c.286+2T>G RV, indicating that they share a circulating defect that results in complement dysregulation on the endothelium. These results highlight the complexity of the genetics of aHUS and indicate that *CD46* deficiency may not be enough to induce aHUS. We hypothesize that the proband inherited from his mother a genetic abnormality in a complement circulating factor that has not been identified yet, which synergized with the *CD46* RV in predisposing him to the aHUS phenotype.

## Introduction

Hemolytic uremic syndrome (HUS) is an ultra-rare disease characterized by microangiopathic hemolytic anemia, thrombocytopenia, and renal impairment (1) caused by platelet thrombi in the microcirculation of the kidney and other organs. Atypical HUS (aHUS) accounts for about 10% of all cases and has a poor prognosis compared with the most common form of HUS in children, which is caused by Shiga-like toxin producing *Escherichia coli* (*STEC*) (1). Before the introduction of C5 inhibition therapy, up to 50% of aHUS cases progressed to end-stage renal failure (ESRF) or developed irreversible brain damage, and 25% died during the acute phase of the disease (2, 3).

The term primary aHUS identifies cases characterized by dysregulation of the alternative complement pathway (2, 4). Rare recessive forms of aHUS are associated with genetically determined cobalamin C (5) or diacylglycerol kinase 3 deficiency (6, 7). Finally, aHUS may be secondary to other conditions, such as autoimmune diseases, systemic diseases, malignant hypertension, and transplantation (4, 8).

In 40–60% of patients with primary aHUS, genetic abnormalities affecting the complement regulatory proteins factor H (*CFH*), membrane cofactor protein (*MCP*), factor I (*CFI*), and thrombomodulin (*THBD*) and the components of the alternative pathway C3 convertase C3 and factor B (*CFB*) or anti-FH autoantibodies have been identified (2, 9–13). Less than 20% of cases are considered familial, that is, cases where two or more members of the same family are affected by the disease and exposure to STEC has been ruled out. All the other patients do not have a family history of the disease (sporadic aHUS), and most of them inherited the complement abnormality from an unaffected parent. Indeed, incomplete penetrance has been reported for all genes associated with aHUS. Other genetic modifiers, including risk haplotypes and polymorphisms and environmental factors have been shown to contribute to the development of disease phenotypes (14–16). A wide variety of triggers have been identified, including common viral and bacterial infections, ischemia, organ transplantation, and pregnancy.

Mutations in the gene encoding membrane cofactor protein (also known as *CD46*), first described in association with aHUS in 2003 (17, 18), account for 5 to 9% of cases with primary aHUS. MCP is a transmembrane protein made by four N-terminal short consensus repeats (SCRs), a serine/threonine-rich (ST) domain, a transmembrane domain (TM), and a cytoplasmic tail (CYT) and serves as cofactor for factor I (FI), a plasma serine protease that cleaves C3b and C4b. It is widely expressed on all nucleated cells and is particularly highly expressed in the kidney (19), where it regulates C3 activation in the glomerulus.

aHUS-associated CD46 variants usually cluster in the extracellular complement regulatory SCR domains (14, 20–22). Seventy-five percent cause a reduction in MCP expression on the cell surface (23, 24).

The penetrance of aHUS among subjects with *CD46* mutations is incomplete, and 25% of patients had combined mutations in other complement genes (12).

Here, we investigated the determinants of *CD46* mutation penetrance. We found that the splicing variant c.286+2T>G (also known as IVS2+2T>G; dbSNP: rs769742294) is the most prevalent *CD46* genetic abnormality in our cohort of patients (*n* = 485) with primary aHUS, and within families, disease penetrance in c.286+2T>G carriers was 28%. We performed an in-depth study of the large pedigree of a patient with sporadic aHUS who is heterozygous for this variant. Fremeaux-Bacchi et al. demonstrated that this splice-site variant results in abnormal splicing, causing a deletion of 48 amino acids in the SCR1 of the protein (24). Afterwards, Maga et al. reported that the c.286+2T>G results in another abnormally spliced mRNA, leading to a frameshift and the translation of a truncated protein (p.Glu97Lysfs^*^33) (25).

In published studies, the c.286+2T>G variant was associated with a variable phenotype: heterozygous carriers have a milder form of aHUS, while the disease is more severe and has an earlier onset in homozygous carriers (24, 26). In contrast, in our pedigree, the heterozygous proband manifested a severe form of aHUS and developed ESRF, while two adult relatives (his father and paternal uncle) with the same variant in homozygosity are unaffected. Screening for other known aHUS-associated complement genes did not reveal rare or common variants that segregated with the disease. An *ex vivo* test showed excessive complement deposition on endothelial cells exposed to sera from the proband, and also from his mother and his maternal uncle, who do not carry the c.286+2T>G variant, indicating that the proband inherited a maternal circulating defect in complement regulation. These results highlight the complexity of the genetics of aHUS and indicate that CD46 deficiency may not be not enough to cause aHUS.

## Materials and Methods

### Study Subjects

Four hundred eighty-five unrelated patients with a diagnosis of primary aHUS were recruited through the International Registry of HUS/TTP, under the coordination of the Aldo and Cele Daccò Clinical Research Center for Rare Diseases (Ranica, Bergamo, Italy).

Clinical information and demographic and laboratory data for all patients and their available relatives were collected using a case report form. Biochemical and genetic tests were performed on blood, plasma, serum, and DNA samples collected from all aHUS patients and available relatives.

All participants received detailed information on the purpose and design of the study, according to the guidelines of the Declaration of Helsinki.

aHUS was diagnosed in all cases with microangiopathic hemolytic anemia and thrombocytopenia [defined as hematocrit (Ht) <30%, hemoglobin (Hb) <10 g/dl, serum lactate dehydrogenase (LDH) of >500 IU/L, undetectable haptoglobin, fragmented erythrocytes in the peripheral blood smear, and platelet count less of than 150,000/μl] associated with acute renal failure (serum creatinine >1.3 mg/dl for adults, >0.5 mg/dl for children under 5 years old and >0.8 mg/dl for children aged 5–10, and/or urinary protein–creatinine ratio >200 mg/g; or an increase in serum creatinine or urinary protein–creatinine ratio >15% compared with baseline levels). Thrombotic thrombocytopenic purpura was ruled out because all patients exhibited ADAMTS13 activity >10% and no anti-ADAMTS13 antibodies. Primary aHUS was defined in aHUS patients when both secondary underlying conditions and infections by Stx-*E.coli* were ruled out.

Ethnically matched healthy controls (*n* = 319) were also recruited from blood donors and were screened for *CD46* variants that we found in primary aHUS patients.

All participants provided informed written consent. The study protocol was approved by the Ethics Committee of the Azienda Sanitaria Locale, Bergamo, Italy.

### Genetic Screening and Biochemical Testing

Genomic DNA was extracted from peripheral blood leukocytes (Nucleon BACC2 kit, Amersham; NucleoSpin Blood kit, Macherey-Nagel). All coding exons and the intronic flanking regions of membrane cofactor protein (*CD46*), complement factor H, (*CFH*), complement factor I (*CFI*), complement factor B (*CFB*), complement C3 (*C3*), and thrombomodulin (*THBD*) genes were amplified by polymerase chain reaction (PCR), using gene-specific primers and standard conditions. Amplification products were sequenced using standard Big-Dye Terminator v.1.1 protocols on 48-capillary 3730 DNA Analyzer. Sixty patients who were recruited more recently were analyzed using a home-made next-generation sequencing (NGS) diagnostic mini panel for simultaneous sequencing of the six complement genes reported above using a combination of multiplex PCR and high-throughput sequencing (PGM Ion Torrent, Life technologies). dbSNP, 1,000 genomes, ESP6500, and ExAC databases were used to distinguish new variants from those that had already reported. Ethnically matched healthy controls (n = 319) were screened for rare *CD46* variants found in non-Stx-HUS patients.

Genetic variants with a reported minor allelic frequency (MAF) below 0.001 in 1,000 Genomes and in the Exome Aggregation Consortium (ExAC), not found in 319 healthy controls and with a Combined Annotation Dependent Depletion (CADD) phred score ≥ 10 were considered rare variants (RVs). *CD46* RVs were further classified in “pathogenic,” “likely pathogenic,” “uncertain significance,” “likely benign,” or “benign” using the guidelines from the American College of Medical Genetics and Genomics (ACMG) (27) and Kidney Disease: Improving Global Outcomes (KDIGO) conference (4).

Once a RV was identified, the parents and relatives of the proband were invited for genetic testing to study the transmission model.

In patients carrying the *CD46* c.286+2T>G RV and their available relatives, we genotyped by direct sequencing the *CFH* single-nucleotide polymorphisms (SNPs) (c.1-331C>T, rs3753394; c.184G>A, p.V62I, rs800292; c.1204C>T, p.H402Y, rs1061170; c.2016A>G, p.Q672Q, rs3753396; c.2237-543G>A, rs1410996; c.2808G>T, p.E936D, rs1065489) that define the disease risk haplotype *CFH*_*TGTGGT*_ (known as *CFH–H3* haplotype) and one SNP in *CD46* (rs7144, c.^*^897T>C) that tags the risk *CD46*_*GGAAC*_ haplotype.

All *CFHR5* exons and the three nucleotide differences in exon 4 of *CFHR1* (c.469, c.475, and c.523) that distinguish the CFHR1^*^A and CFHR1^*^B alleles were also genotyped in the proband and his relatives, using direct sequencing. The three differences cause three amino acid changes in SCR3 of FHR1 that make SCR3 of FHR1 identical to SCR18 of FH (28). The SCR3 with Tyr157, Val159, and Gln175 amino acids characterizes the basic isoform of FHR1 and is identical to the SCR18 of FH, indicating that the CFHR1^*^B allele could be the result of a gene conversion between *CFH* and *CFHR1*. The CFHR1^*^B allele has been found to be associated with aHUS patients carrying the homozygous CFHR1^*^B allele.

Multiplex ligation-dependent probe amplification (SALSA MLPA P236-A3 ARMD, MCR-Holland) was used to evaluate copy number variations in *CFH, CFHR3, CFHR1, CFHR2*, and *CFHR5* genes.

The mRNA extracted from peripheral blood mononuclear cells (PBMCs) from the proband and his relatives in family #646 was reverse transcripted using SuperScript® II Reverse Transcriptase (Invitrogen, Carlsbad, CA, United States). cDNA amplification and sequencing were performed using a forward primer constructed on exon 1 (signal peptide) (5′-GCTTTCCTGGGTTGCTTCT) and a reverse primer constructed on exon 3 (SCR2) (5′-CATTTGCAGGGACTGCTTG).

Complement C3 and C4 serum levels were evaluated using kinetic nephelometry (14). Plasma SC5b-9 levels were evaluated with the MicroVue SC5b-9 Plus EIA commercial kit (SC5b-9 Plus, Quidel). The presence of anti-FH antibodies was evaluated using enzyme-linked immunosorbent assay (ELISA) (13).

### CD46 Expression Studies on Peripheral Blood Mononuclear Cells

PBMCs were isolated from the peripheral blood of the proband, his relatives, and healthy volunteers by performing density gradient centrifugation using Ficoll-Paque. Fresh or thawed PBMCs were labeled with anti-human CD3 APC-Cy7 or alternatively anti-human CD3 BV510 (clone SK7) and with antibody anti-human CD46 fluorescein isothiocyanate (FITC) (clone E4.3 that recognizes SCR1 epitope) or with antibody anti-human CD46 FITC (clone MEM258 that recognizes SCR4 epitope). Each experiment was performed using PBMCs from healthy volunteers labeled for CD3 and CD46 (SCR1) or CD3 and CD46 (SCR4) or unlabeled as negative controls (29). The gating strategy is reported in Supplementary Figure 1.

Samples were acquired with FACSAria or LSR-Fortessa X-20 cytofluorimeter (BD) and analyzed using FlowJo software (BD).

Samples were labeled with the VIABILITY dye probe to exclude dead cells from analysis 10 min before acquisition.

FMO (Fluorescence minus one) was used to analyze samples. Samples were gated in single cells, and T live cells were then analyzed for the specific expression of CD46 [as median fluorescence intensity (MFI)] for SCR1, or for SCR4. CD46 expression was indicated as MFI percentage compared with the control.

### Complement Deposition on Human Microvascular Endothelial Cells

A human microvascular endothelial cell line of dermal origin (HMEC-1) was plated on glass slides and used when confluent. Cells were activated with 10 μM of adenosine 5′-diphosphate (ADP) for 10 min and then incubated for 4 h with serum diluted 1:2 with test medium [Hanks' Balanced Salt Solution (HBSS) with 0.5% bovine serum albumin (BSA)]. At the end of the incubation step, HMEC-1 were stained with FITC-conjugated rabbit anti-human C3c-complement or rabbit anti-human complement C5b-9 followed by FITC-conjugated secondary antibody. In each experiment, a pool of sera from healthy controls was tested parallel with the patient's serum. We verified the cellular integrity after exposure to serum samples in parallel slides in which HMEC-1 were stained with May-Grunwald Giemsa (30). A confocal inverted laser microscope was used for the acquisition of the fluorescent staining on the endothelial cell surface. Fifteen fields per sample were acquired, and the area with fluorescent staining was evaluated with automatic edge detection using built-in functions in the Image J software and expressed as pixel^2^ per field analyzed. The fields with the lowest and highest values were excluded from calculation. Results were expressed as percentage of staining compared with control serum pool.

### Statistical Analysis

MedCalc software was used for statistical tests. Fisher's exact test was used to compare the frequency of *CD46* RVs between aHUS patients and healthy control populations. Chi-square test or Fisher's exact test was used to calculate the risk of *CFH–H3* and *CD46*_*GGAAC*_ haplotypes to increase the risk of developing aHUS. The results of C5b-9 deposition on activated HMEC were expressed as mean ± SE and analyzed by ANOVA.

*P*-values of less than 0.05 were considered statistically significant.

## Results

### c.286+2T>G Is the Most Frequent *CD46* Rare Variant in Atypical Hemolytic Uremic Syndrome and Is Associated With Incomplete Penetrance

Screening of complement disease-associated genes in 485 unrelated patients with primary aHUS (herein defined aHUS) identified RVs in 189 cases. Specifically, 17% of patients carry *CFH* RVs; 7% *CFH–CFHR* rearrangements; 8% RVs in *CD46*; and 4, 8, 2, and 1% in *CFI, C3, CFB*, and *THBD* genes, respectively. In addition, in 10% of patients, we identified anti-FH antibodies.

We identified 15 *CD46* RVs in 39 patients. Of these, 20% (*n* = 8) exhibited additional RVs in *CD46* (*n* = 3) and/or in other complement genes (*n* = 5) (Table 1). Nonsense and frameshift RVs were found in 13 aHUS patients, whereas no non-sense or frameshift RVs were found in any of the 319 healthy controls (13/485 vs. 0/319; *P* = 0.002).

**Table 1:** List of *CD46* rare variants (RVs; variants with minor allele frequency < 0.001 in 1000 Genomes and ExAC databases and with CADD phred score ≥10) identified in 485 atypical hemolytic uremic syndrome (aHUS) patients recruited through the International Registry of HUS/TTP.

**Exon**	***CD46* rare variants**	**Patients (*n* = 485)**	**Ctrs (*n* = 319)**	***P*-value**	**dbSNP rs**	**HGMD**	**1,000 K frequency**	**ExAC global frequency**	**Pathogenic in functional studies**	**CADD**	**Variant classification**
Intron 1	c.98-1G>C (p.C35X)	4	0	0.16	rs1441937053	CS064376	NA	NA	Yes^1^	24.1	P
Exon2	c.104G>A (p.C35Y)	3^a^	0	0.28	rs121909591	CM062498	NA	8.26 × 10^−6^	Yes^1^	24.5	LP
Exon2	c.175C>T (p.R59X)	6^b^	0	0.09	rs121909590	CM062495	NA	NA	Yes^1^	24.5	P
Exon2	c.192_198delinsC (p.C64fs)	1	0	1.00	NA	CX064751	NA	NA	NA	22.5	LP
Intron 2	c.286+2T>G	13^c^	0	*0.002*	rs769742294	CS066620	NA	3.3 × 10^−5^	Yes^2^	23.7	P
Intron 2	c.287-2A>G	3^d^	0	0.28	rs759813089	CS064377	NA	3.1 × 10^−5^	Yes^1^	23.9	LP
Exon3	c.295T>C (p.C99R)	1	0	1.00	NA	CM062496	NA	NA	Yes^1^	23.8	P
Exon3	c.307C>T (p.R103W)	1	0	1.00	rs1486782648	CM050660	NA	NA	Yes^3^	10.3	US
Intron 4	c.475+1_4delGTAA	1^e^	0	1.00	NA	CD136074	NA	NA	NA	16.8	NA
Exon 5	c.565T>G (p.Y189D)	4^f^	0	0.16	rs202071781	CM103418	1.99 × 10^−4^	8.2 × 10^−6^	Yes^2^	23.8	LP
Exon 5	c.648G>C (p.W216C)	1	0	1.00	NA	CM103419	NA	NA	NA	24.9	NA
Exon 6	c.685C>T (p.R229X)	1	0	1.00	rs1553251787	CM1516829	NA	NA	NA	35	LP
Exon 6	c.799-800delAC (p.T267fs)	1	0	1.00	NA	CD034158	NA	NA	Yes^4^	15.03	P
Exon 6	c.815_832delinsATT+ c.841C>A (p.272-276del+D277N+P278S)	1	0	1.00	NA	CX064752	NA	NA	Yes^1^	25.4	NA
Exon 6	c.725T>G (p.F242C)	1^g^	0	1.00	NA	CM062499	NA	NA	Yes^1^	22.3	P

*Nucleotide CD46 numbering was used according to GenBank sequence NM_002389.4. Statistical analysis was performed using Fisher's exact test. Statistical significance: P < 0.05. Italic characters indicate statistically significant values*.

*HGMD, Human Gene Mutation Database; 1000 K, 1000 Genome Project; ExAC, Exome Aggregation Consortium; CADD, Combined Annotation Dependent Depletion; Variant Classification reported in the Database on complement gene variant (https://www.complement-db.org/home.php) based on guidelines from ACMG and Goodship et al. (4); P, pathogenic; LP, likely pathogenic; US, uncertain significance; NA, not available*.

a *A patient (#F106) carries two additional RVs (CD46 p.R59X; FH p.R1210C)*.

b *Two patients have combined complement RVs: patient #1075 also carries a THBD pathogenic variant (p.A43T); patient #F106 carries two additional RVs (CD46 p.C35Y; FH p.R1210C)*.

c *Four patients have additional complement variants: patient #S978 also carries a FI (p.I357M) and an FH (p.R1210C) RVs; patient #1503 carries an additional RV in CD46 (p.Y189D). Patient #1873 also carries an FH RV (p.N516K). Patient #1793 also carries a FI RV (p.R317Q)*.

d *A patient (#657) also carries a FI RV (p.L484Vfs3X)*.

e *This patient (#1430) carries an additional CD46 RV (p.Y189D)*.

f *Two patients (#1430 and #1503) also carry additional complement RVs (#1430: c.475+1_delGTAA; #1503: c.286+2T>G)*.

g *This patient (#F169) also carries an FH RV (p.G1194D)*.

1 *Caprioli et al. (23)*.

2 *Fremeaux-Bacchi et al. (24)*.

3 *Fang et al. (31)*.

4 *Noris et al. (17)*.

c.286+2T>G is the most frequent RV that we identified in aHUS patients of our Registry and is significantly overrepresented in patients compared with controls (patients 13/485 vs: healthy controls 0/319, *P* = 0.002; ExAC controls 4/60412, *P* = 1 × 10^−6^) (Table 1). Patients carrying this RV had an age of onset ranging from 1 to 58 years. Through the genetic analysis of seven pedigrees, we found 25 subjects carrying the c.286+2T>G variant. Given that only seven individuals in the above pedigrees developed the disease, the penetrance of aHUS in c.286+2T>G carriers is 28%.

**Figure 1:**
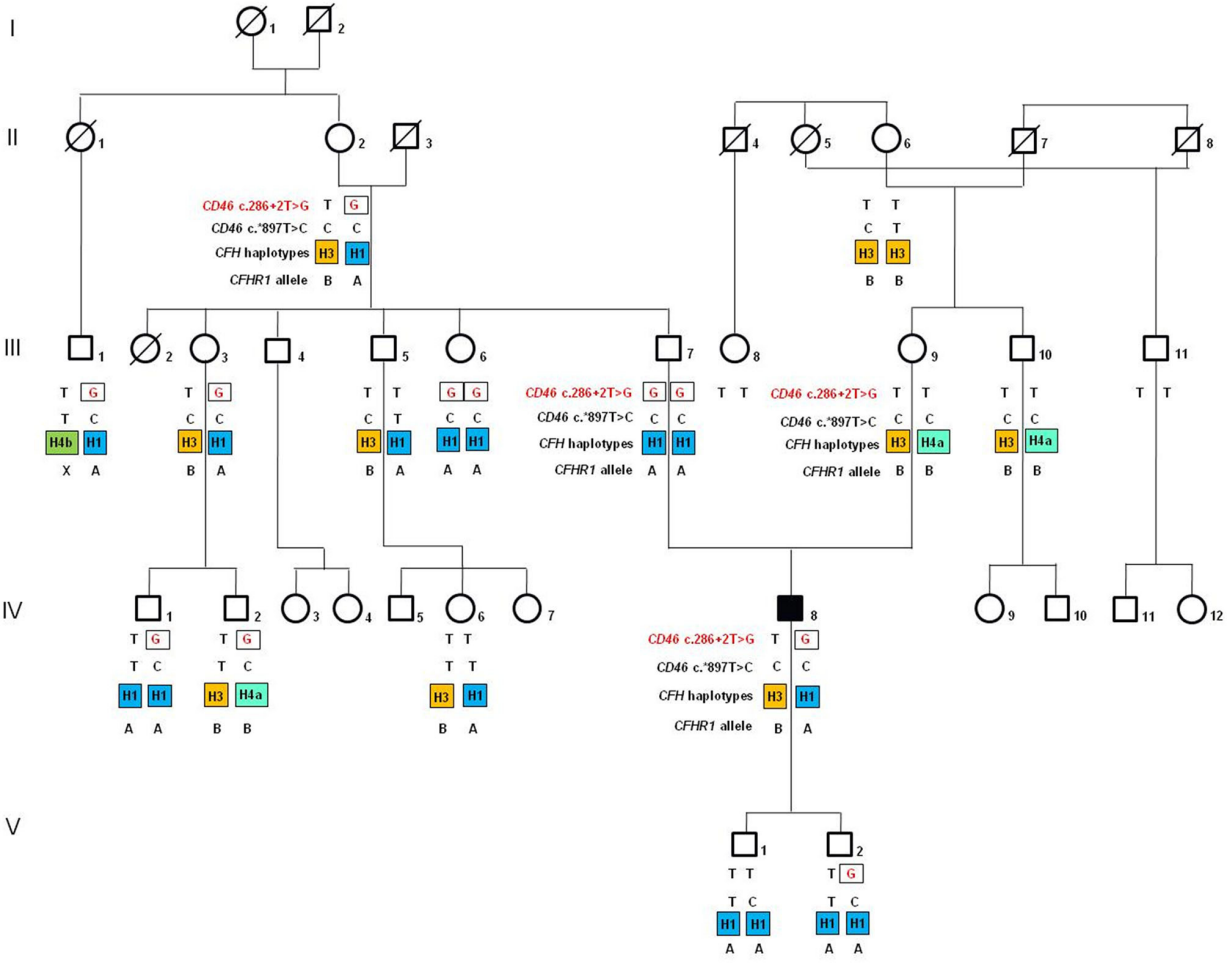
Pedigree (#646) of the atypical hemolytic uremic syndrome (aHUS) patient (IV-8; indicated by the black square) carrying the heterozygous c.286+2T>G rare variant (rs769742294) in the gene encoding membrane cofactor protein (*CD46*). The *CD46* c.286+2G rare variant is indicated in red. The *CD46* c.*897T>C SNP (rs7144) tags the risk *CD46*_*GGAAC*_ haplotype. In orange is indicated the disease risk haplotype *CFH*_*TGTGGT*_ (*CFH−H3* haplotype), while in blue and in green are indicated *CFH* haplotypes (*CFH*_*CGGAGG*_ or *CFH-H1*; *CFH*_*CGTAAG*_ or *CFH-H4a*; *CFH*_*TGTAAG*_ or *CFH-H4b*) not associated with aHUS. A indicates the CFHR1*A allele, which encode the acid (A) isoform of FHR1, and B indicates the CFHR1*B allele that is a risk factor for aHUS when present in homozygosity and encodes the basic (B) isoform of FHR1.

**Figure 2:**
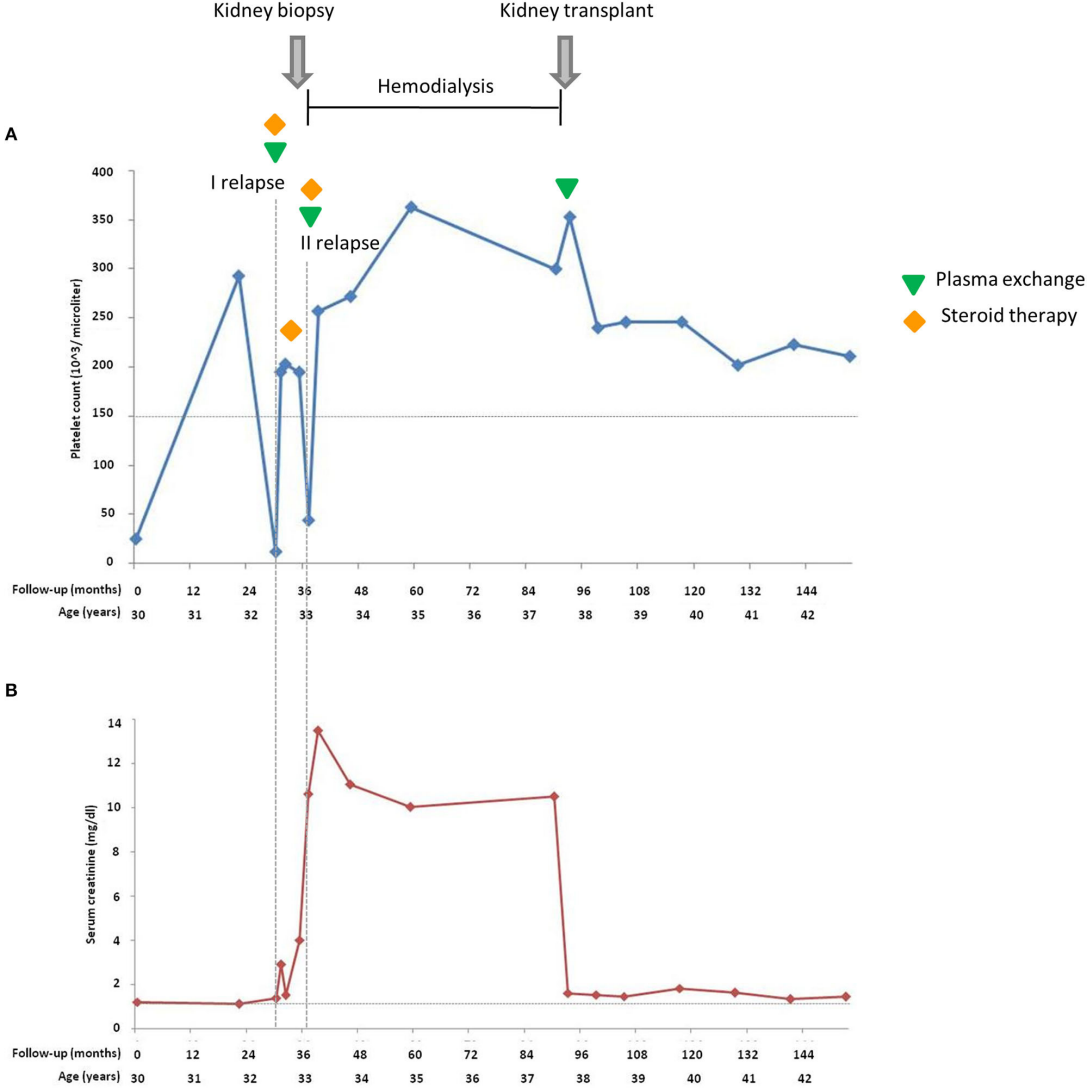
Platelet count **(A)** and serum creatinine **(B)** of the proband during 153 months of follow-up from the atypical hemolytic uremic syndrome (aHUS) onset. *Horizontal dashed lines* in **(A)** indicate the lower limit of normal platelet count (150,000/μl); in **(B)** indicate the upper limit of the normal serum creatinine range. *Vertical dashed lines* highlight the two relapses.

Among the seven studied pedigrees, we further focused on the large family identified as #646 as a prototype of incomplete penetrance associated with the c.286+2T>G variant (Figure 1). In this pedigree, the proband was born from non-consanguineous, healthy parents. After an episode of acute gastroenteritis at 29.5 years of age, the man developed hypertension, jaundice, and thrombocytopenia (platelet count 25,000/μl) with borderline serum creatinine levels (1.2 mg/dl) and normal Hb levels (15 g/dl). The episode resolved spontaneously, platelet count normalized (293,000/μl), and Hb levels and renal function were normal (16.6 g/dl and 1.1 mg/dl, respectively) (Figure 2). At age 32, he was hospitalized with a fever and jaundice. Laboratory results revealed a low platelet count (12,000/μl, Figure 2), an increased LDH level (1,619 U/L), hyperbilirubinemia, fragmented red cells (schistocytes), and slightly elevated serum creatinine levels (1.36 mg/dl). A clinical diagnosis of aHUS was made. After 10 plasma exchanges and oral therapy with high doses of steroids (1 mg/kg), the hematologic features improved but renal function worsened (creatinine levels up to 2.9 mg/dl, Figure 2). The patient continued steroid therapy, leading to improvement in renal function (serum creatinine: 1.5 mg/dl) and an increase in platelet numbers (203,000/μl, Figure 2). After 3 months, serum creatinine was 4 mg/dl (Figure 2), and the patient underwent a kidney biopsy, which showed thrombotic microangiopathy with ischemic nephropathy. Two months later, he had an aHUS relapse with severe thrombocytopenia (44,000/μl) severe renal failure (serum creatinine 10.6 mg/dl, Figure 2) and lower than normal C3 and C4 levels (44 and 4 mg/dl, respectively). He was treated with plasma exchange and steroid therapy and underwent hematological remission but developed ESRF requiring chronic hemodialysis. One year later, C3 and C4 serum levels were normal, Hb levels were borderline (between 11 and 11.9 g/dl), and the platelet count was normal (Table 2).

**Table 2:** Clinical, complement, genetic, and CD46 expression data in the patient and his asymptomatic relatives.

				**Genetic data**	**Clinical parameters**				**Complement parameters**					**CD46 protein expression**^**†**^	
**Disease status**	**Subject**	**Gender**	**Age (years)^a^**	***CD46*: c.286+2T>G**	**Platelets (×10^**3**^/μl)^b^**	**LDH (IU/L)^c^**	**Hb (g/dl)^d^**	**sCr (mg/dl)^e^**	**C3 (mg/dl)^f^**	**C4 (mg/dl)^g^**	**sC5b-9 (ng/ml)^h^**	**C3 deposits^i^ (% of control)**	**C5b-9 deposits^i^ (% of control)**	**With anti-SCR1 antibody (% of control)**	**With anti-SCR4 antibody (% of control)**
Affected	IV-8	M	34	TG	363	434	11.9	10.03	114	47	250.7	380	464	46	63
Unaffected	III-7	M	57	GG	204	392	14.6	0.83	nd	nd	136.2	135	104	7	15
Unaffected	III-9	F	52	TT	226	334	13.1	0.76	nd	nd	140.1	275	358	95	100
Unaffected	II-2	F	82	TG	209	454	13.4	1.13	nd	nd	nd	nd	nd	37	54
Unaffected	III-6	F	49	GG	277	376	12.4	0.56	nd	nd	nd	nd	nd	8	9
Unaffected	III-3	F	60	TG	151	401	13.4	0.81	nd	nd	nd	nd	nd	40	56
Unaffected	III-5	M	50	TT	151	437	14.1	0.81	nd	nd	nd	nd	nd	91	100
Unaffected	V-1	M	7	TT	215	nd	13.5	0.47	nd	nd	nd	nd	nd	97	nd
Unaffected	III-1	M	51	TG	245	482	16	0.76	nd	nd	211.5	nd	103	nd	nd
Unaffected	III-10	M	55	TG	nd	334	nd	1.08	nd	nd	nd	193	337	nd	nd

a *At this age, the proband was in hematological remission and chronic hemodialysis. Age of relatives refers to the data of the visit done at our center for genetic, clinical, and complement CD46 expression analyses*.

*Abbreviations and limit of normal range*.

b *150–400 × 10^3^/μl*.

c *LDH, lactate dehydrogenase: 266–500 IU/L*.

d *Hb, hemoglobin: 14–18 g/dl (men), 12–16 g/dl (women)*.

e *sCr, serum creatinine: 0.55–1.25 mg/dl*.

f *C3: 79–170 mg/dl*.

g *C4: 10–40 mg/dl*.

h *Normal plasma sC5b9 levels: ≤400 ng/ml*.

i *Expressed as percentage of control. Limit of normal range < 150%*.

*CD46 protein levels in CD3+ positive peripheral blood mononuclear cells (PBMCs). Values are expressed as percentage median fluorescence intensity in control CD3+ PBMCs. Data are mean of results of 2/3 experiments*.

*nd, not done*.

At age 37, the patient underwent a cadaveric renal transplantation with prophylactic plasma exchange to prevent post-transplant recurrence (Figure 2). The patient received an induction treatment with low doses of thymoglobulin and basiliximab, and maintenance immunosuppression with steroids, cyclosporine, and azathioprine. Hypertension was treated with atenolol and nifedipine. On day 9 post-transplant, the platelet count and Hb level were normal, and serum creatinine was 1.6 mg/dl. At the last follow-up, 5 years after transplantation, blood pressure was normal (136/72 mmHg), serum creatinine was 1.43 mg/dl, and the platelet count and Hb levels were within normal ranges, while proteinuria was absent (0.08 g/24 h).

The proband carries the *CD46* c.286+2T>G RV in heterozygosity. The family history is negative for aHUS and other kidney diseases. Genetic analysis for the c.286+2T>G variant was extended to 16 healthy relatives. As shown in Figure 1 (pedigree #646), the c.286+2T>G variant is of paternal origin. Surprisingly, the proband's unaffected father (III-7) and one paternal aunt (III-6) are homozygous for the c.286+2T>G RV. Furthermore, six paternal relatives are healthy carriers of the same variant in heterozygosity (Figure 1).

Sequencing of other complement aHUS-associated genes (*CFH, CFI, C3, CFB, THBD*, and *CFHR5*) in the proband and his parents did not reveal additional RVs. The proband has a normal copy number of *CFH–CFHR* genes. The assay for anti-FH antibodies that was done before transplantation showed negative results.

We then investigated whether genetic modifiers previously associated with aHUS, including the *CFH–H3* and the *CD46*_*GGAAC*_ haplotypes (32–35), and the CFHR1^*^B allele (28) could account for disease penetrance in family #646. The proband is heterozygous for the *CFH* SNPs of the risk H3 haplotype (*CFH*_*TGTGGT*_), which he inherited from his mother. In addition, he is homozygous for the c.^*^897T>C SNP (rs7144), which tags the *CD46*_*GGAAC*_ risk haplotype and was inherited from both parents, who are homozygous for this SNP. Finally, he is heterozygous for the CFHR1^*^B allele (Figure 1).

Unlike the proband, his father does not carry the risk *CFH–H3* haplotype. Indeed, he is homozygous for the neutral *CFH-H1*, previously found with the same frequency in aHUS patients and in the control population (34, 36). In addition, the father does not carry the CFHR1^*^B allele. However, as shown in Figure 1, two unaffected adult paternal relatives who are heterozygous for c.286+2T>G RV (II-2, the paternal grandmother and III-3, a paternal aunt; 83 and 49 years old, respectively) share the same risk factors as the proband. Indeed, they have one copy of *CFH–H3*, two copies of the *CD46*_*GGAAC*_, and one copy of the CFHR1^*^B. These data indicate that the presence of *CFH* and *CD46* risk haplotypes and CFHR1^*^B alleles is not enough to explain the disease in c.286+2T>G carriers.

We extended the analysis of *CFH* and *CD46* risk haplotypes to the other aHUS patients and their available unaffected relatives carrying the *CD46* c.286+2T>G RV. We found no significant association between the presence of *CFH–H3* (*P* = 0.29) or *CD46*_*GGAAC*_ (*P* = 0.12) haplotypes and the disease (Table 3), confirming the results obtained in pedigree #646.

**Table 3:** Analysis showing that *CFH–H3* and *CD46*_*GGAAC*_ haplotypes are not associated with *CD46* c.286+2T>G rare variant.

	***CFH–H3*** **haplotype**				***CD46*** c.^*^897T>C			
**Status**	**het**	**hom**	**No *CFH H3-*haplotype**	**Total subjects screened**	**TT**	**TC**	**CC**	**Total subjects screened**
Affected	3	3	3	9	0	1	10	11
Unaffected	7	1	5	13	0	6	7	13
	*P* = 0.293				*P* = 0.124			

Patients and relatives carrying the CD46 c.286+2T>G rare variant were genotyped for CFH single-nucleotide polymorphisms (SNPs) and for the CD46 SNP (rs7144; c.^*^897T>C) tagging the CD46_GGAAC_ haplotype.

### In Pedigree #646, the *CD46* c.286+2T>G Rare Variant Causes the Formation of Two Abnormal mRNA Variants, Leading to Reduced Cell Surface Protein CD46 Expression

The c.286+2T>G RV is in the donor splice site of exon 2 of CD46 coding for short consensus repeat 1 (SCR1), which is one of the four extracellular complement regulatory domains of the protein. The variant has already been described as affecting exon 2 splicing (24, 25). In 2006, Fremeaux-Bacchi et al. (24) described that it results in aberrant mRNA that lacks 144 bp of exon 2 and encodes a protein that is missing 48 amino acids in the SCR1 domain in phase with the wild-type protein sequence. In 2010, Maga et al. (25) provided different results and showed that the c.286+2T>G RV leads to a frameshift at p.R96 (SCR2) of the protein, with the addition of several amino acids, followed by a premature truncation at position p.129X (SCR2).

**Figure 3:**
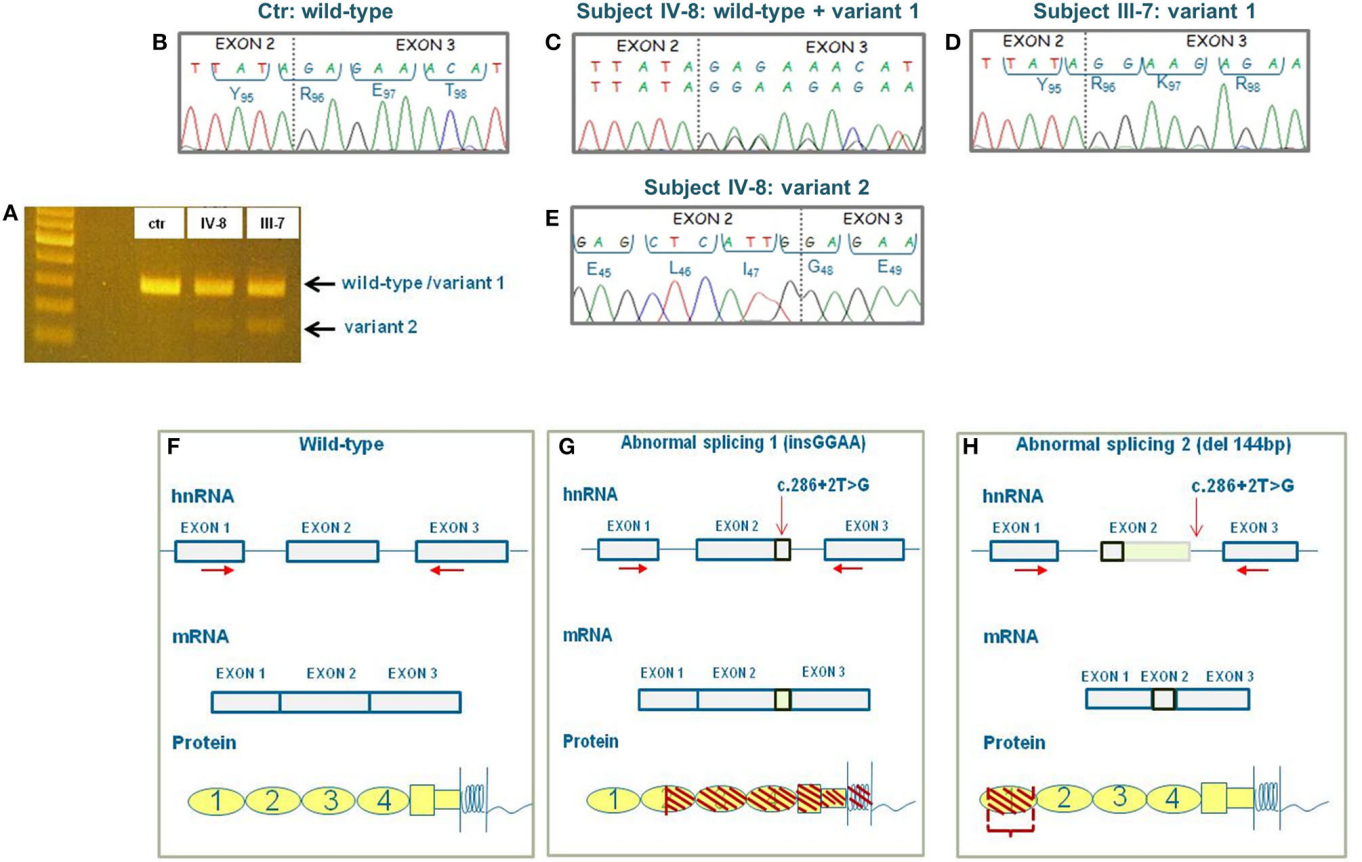
Results of CD46 cDNA sequencing and prediction of the effect of the abnormal splicings at protein level. **(A)** Agarose gel image of cDNA showing a single band in the control (ctr) and two bands in the proband (IV-8) and in his healthy father (III-7). First lane: 100-bp ladder used as a size marker. **(B)** Electropherogram showing the wild-type cDNA sequence of the control. **(C)** Electropherogram of the upper band in the proband (subject IV-8) showing the wild-type sequence and the variant 1's sequence with 4-bp insertion. **(D)** Electropherogram of the upper band in the homozygous proband's father (subject III-7) showing the sequence of variant 1 only. **(E)** Electropherogram of the lower minor band in the proband showing the sequence of variant 2 with the deletion of 144 bp. **(F)** Wild-type CD46 heterogeneous nuclear RNA (hnRNA), messenger RNA (mRNA), and protein. **(G)** Representation of the effect of splicing variant 1 on CD46 hnRNA, mRNA and protein. The predicted protein is truncated in the SCR2 domain and includes only the first 128 amino acids. **(H)** Representation of the effect of splicing variant 2 on CD46 hnRNA, mRNA, and protein. The predicted protein lacks 48 amino acids of SCR1. Horizontal red arrows indicate the localization of primers used for cDNA sequencing. The vertical red arrow indicates the localization of c.286+2T>G variant.

To clarify the effect of the c.286+2T>G variant in pedigree #646 and to evaluate whether there was any difference in the abnormal splicing between the proband and the unaffected carriers, we extracted RNA from PBMCs from the proband and his relatives. cDNA amplification using the forward primer located on exon 1 and reverse primer located on exon 3 revealed two different bands on agarose gel electrophoresis in the proband and in all carriers (Figure 3A). Sequencing of the two bands revealed the wild-type sequence and two variants in the heterozygous proband, as well in the heterozygous healthy relatives. The upper band includes both the wild-type sequence and a second sequence (variant 1) with a 4-bp insertion, which corresponds to the variant described by Maga et al. (25) (control: Figure 3B, proband: Figure 3C). cDNA sequence analysis of the upper band from two homozygous relatives showed the variant 1 sequence, but not the wild-type sequence (Figure 3D). The sequence of the minor band (variant 2) corresponds to the splicing variant with the 144-bp deletion described by Frémeaux-Bacchi et al. (24) (Figure 3E). These data demonstrate that (1) the two aberrant mRNA splicing isoforms coexist both in the proband and in his unaffected carrier relatives; (2) variant 1 (with the 4-bp insertion) is the predominantly expressed variant both in heterozygous and homozygous carriers; and (3) in the homozygous unaffected carriers, no appreciable wild-type mRNA is transcribed. Thus, the characterization of abnormal RNAs and their proportion in the pedigree do not explain the incomplete penetrance of the c.286+2T>G RV in this pedigree.

**Figure 4:**
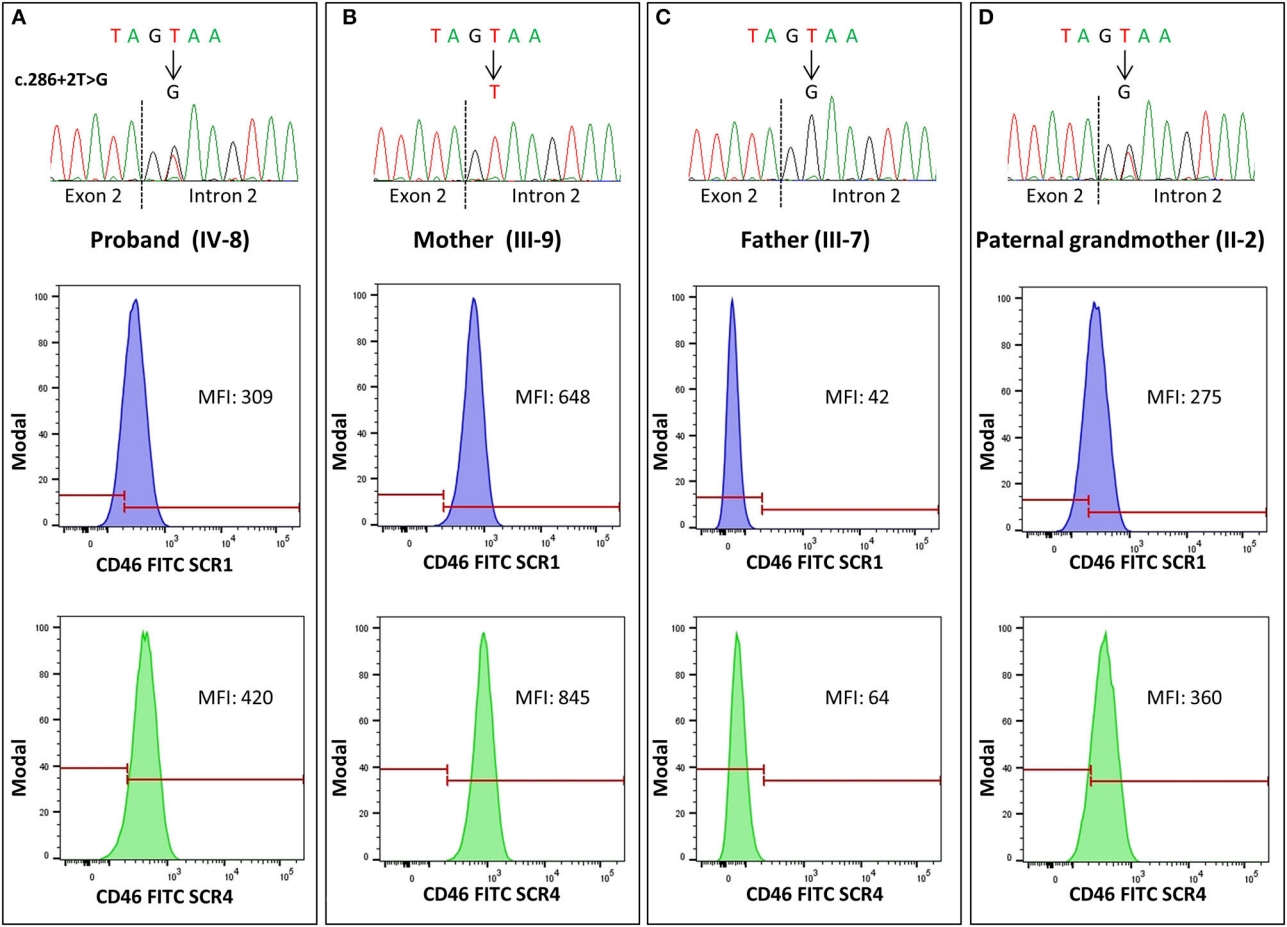
Representative histograms of flow cytometry analysis in pedigree #646, performed with anti-SCR1 and anti-SCR4 antibodies showing levels of CD46 on CD3+ peripheral blood mononuclear cells (PBMCs), expressed as median fluorescence intensity (MFI). The heterozygous carriers of the 286+2T>G rare variant [the proband IV-8, **(A)**, and the paternal grandmother II-2, **(D)**] showed about 50% CD46 expression compared with the wild-type mother [subject III-9, **(B)**], while in the homozygous patient's father [subject III-7, **(C)**], a severe reduction of CD46 expression (<8% compared with the wild-type mother) was observed. The electropherograms of DNA sequences in the proband and his relatives are shown on the top of each panel.

The predicted effect of the two aberrant mRNA splicing isoforms on protein sequence in respect to wild-type protein is shown in Figures 3F–H. To investigate whether the c.286+2T>G RV affected the levels of CD46 protein on cell surfaces in the proband and in his unaffected carrier relatives differentially, we performed fluorescence-activated cell sorting (FACS) studies in peripheral blood leukocytes (Table 2 and Figure 4). With the use of an anti-SCR1 antibody, the MFI on PBMCs from the proband was 46%, compared with that found on PBMCs from healthy controls (Table 2). Similar levels of MFI were observed on PBMCs from the heterozygous healthy carriers (37% in II-2 and 40% in III-3, Table 2). These results indicate that the c.286+2T>G RV severely impairs cell surface protein expression.

Impressively, the PBMCs from the two healthy homozygous relatives (the father, III-7 and the paternal aunt, III-6) exhibited <8% of normal CD46 expression (Table 2), demonstrating an almost complete CD46 deficiency.

Since the anti-SCR1 antibody cannot recognize the less abundant splicing variant 2 that lacks 48 amino acids in SCR1, we repeated the FACS analysis using an antibody targeting the SCR4 of CD46. CD46 surface expression was about 63% normal in the proband, 54–56% in the heterozygous healthy carriers, and 12% in the homozygous family members (Table 2).

Thus, with both antibodies, CD46 protein expression was similar and close to half-normal levels in the proband and the heterozygous carriers, whereas healthy homozygous relatives had an almost complete CD46 deficiency.

In the wild-type family members, the CD46 expression on PBMCs was similar to that observed in healthy controls with both anti-SCR1 and anti-SCR4 antibodies (Table 2).

Representative FACS histograms are shown in Figure 4.

### In Pedigree #646, a Circulating Defect of Maternal Origin Causes Complement Dysregulation on Endothelial Cells

**Figure 5:**
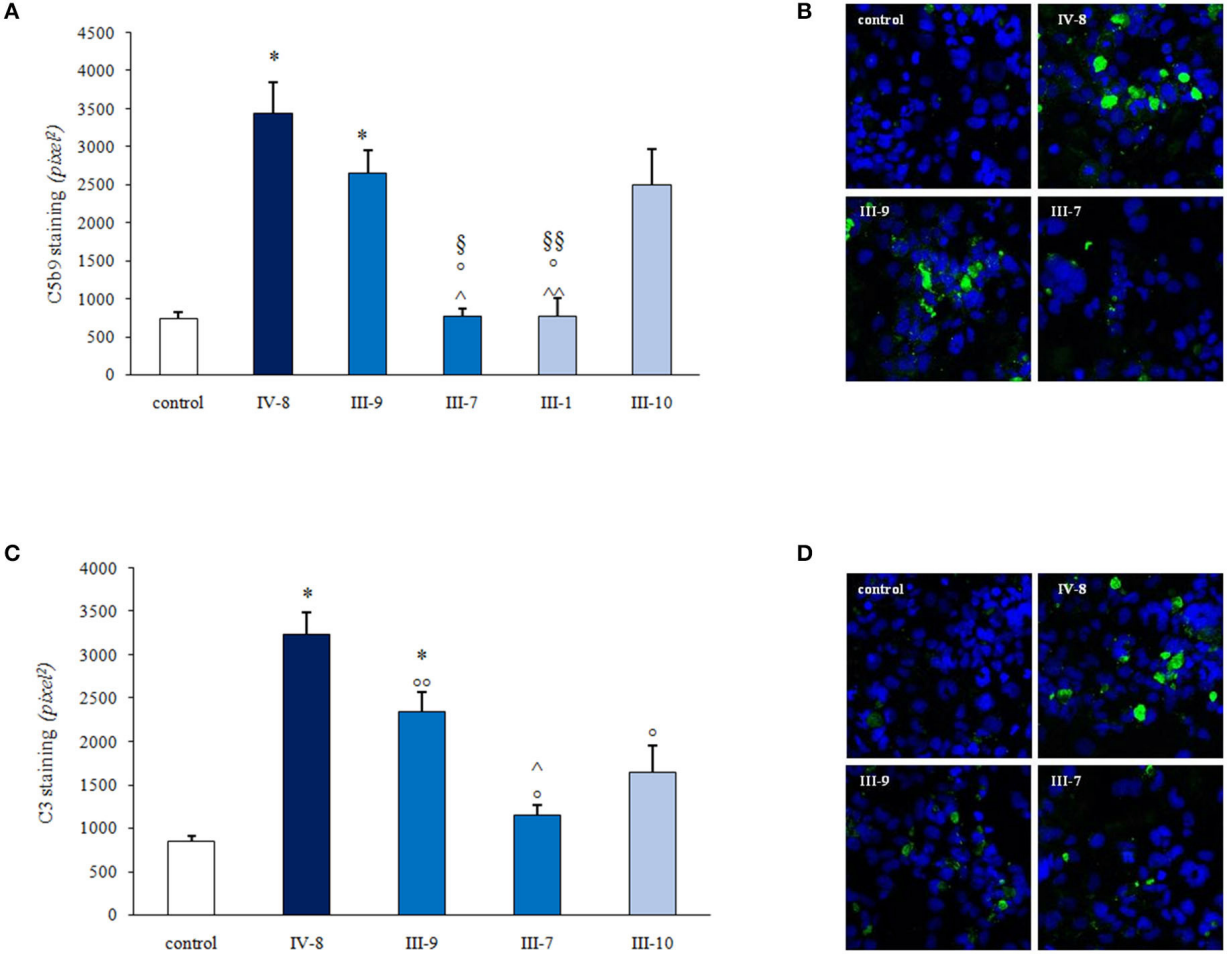
Serum-induced C3 and C5b-9 deposition on human microvascular endothelial cell line (HMEC-1) activated with adenosine 5′-diphosphate (ADP). **(A,C)** Histograms show the quantification of C5b9 **(A)** or C3 **(C)** staining on activated HMEC-1 after 4 h of incubation with the serum from a healthy control (control), the patient (IV-8; heterozygous for *CD46* c.286+2T>G variant), patient's father (III-7; homozygous for *CD46* c.286+2T>G variant), the patient's mother (III-9; with no complement RVs), a paternal relative (III-1; heterozygous for *CD46* c.286+2T>G variant), and a maternal relative (III-10; with no complement RVs). Data are means ± SE; *P < 0.0001 vs. control; °*P* < 0.0001 vs. IV-8; °°*P* < 0.01 vs IV-8; ^∧^*P* < 0.0001, ^∧∧^*P* < 0.001 vs. III-9; ^§^*P* < 0.01, ^§§^*P* < 0.05 vs. III-10. Representative confocal microscopy images show strong C5b-9 **(B)** and C3 **(D)** staining (green) on activated H-MEC1 induced by serum from the patient (IV-8) and from his unaffected mother (III-9) but not by the serum from the proband's father (III-7; **B**,**D**). The blue color indicates the 4′-6-diamidino-2-phenylindole (DAPI) staining of cell nuclei.

The complement system is constituted by over 50 components, and its activation is strictly regulated by circulating and membrane bound proteins to prevent injury to host cells. We hypothesized that a defect in a circulating factor resulting in complement dysregulation at the endothelial cell level could have synergized with *CD46* RV in inducing the disease in the proband. To address this issue, we used a specific *ex vivo* test in which serum from patients with aHUS, studied either during the acute phase or in remission, induces intense deposition of complement products on microvascular endothelial cells (HMEC-1) (37, 38). We exposed ADP-activated HMEC-1 to serum from the proband and from his relatives and evaluated the surface area covered by deposits of the terminal complement complex C5b-9. Serum from the proband (IV-8 in Figure 5), collected while he was in remission when he was on chronic hemodialysis, deposited significantly more C5b-9 on HMEC-1 than control serum (464% of C5b-9 deposits recorded with a pool of sera from healthy controls, Figures 5A,B). Similar results (589%) were obtained with serum collected 2 weeks after kidney transplantation.

Serum from the unaffected father (III-7), who is homozygous for the *CD46* RV, and serum from a heterozygous unaffected relative (III-1) did not increase endothelial C5b-9 deposits (Figures 5A,B). At variance, abnormally high C5b-9 deposits were induced on HMEC-1 by serum from the proband's mother (III-9; 358% of control sera) and from a maternal uncle (III-10; 356%) who do not carry the *CD46* RV (Figures 5A,B). These results indicate that the proband inherited from his mother an abnormality in a circulating factor that predisposes to complement activation on endothelial cells.

Similar results were obtained when we repeated the *ex vivo* test on activated HMEC-1 using an anti-C3c antibody to detect deposits of C3 activation products. Results showed that serum from the proband in remission induced significantly higher C3 deposits than the pool of control sera (380%) (Figures 5C,D). Elevated endothelial C3 deposits were also observed on cells incubated with sera from the proband's mother (III-9, 275%) and the maternal uncle (III-10, 193%) but not with the serum from the proband's father (III-7, 135%) (Figures 5C,D).

## Discussion

Through a retrospective analysis of a cohort of 485 aHUS patients, we performed genetic, molecular, and functional studies to investigate the determinants of penetrance of aHUS associated with genetic abnormalities in *CD46*. This gene encodes a transmembrane glycoprotein that is highly expressed in all tissues, on endothelial cells, and on all circulating cells with the exception of erythrocytes, which regulates both the alternative and classical complement pathways, acting as cofactor for FI to degrade C3b and C4b and to prevent C3 activation on cell surfaces (39).

In this study, *CD46* RVs were found in about 10% of aHUS patients. Consistent with data reported earlier by our group and others, aHUS-associated *CD46* RVs cluster in the extracellular domains of the protein, which are involved in cell surface complement regulation (21, 40).

We focused on the splicing variant c.286+2T>G, which is the most frequent *CD46* RV in our and other aHUS cohorts (21). The c.286+2T>G is also present in the general population, and the allele frequency of this variant in European non-Finnish population is 6.02 × 10^−5^. If this allele were fully penetrant, it would have resulted in 0.012% of population developing aHUS. However, the reported prevalence of aHUS is far lower, ranging between two to ten per million population (0.0002–0.001%) (41), implying that the c.286+2T>G is not enough to develop the disease. Consistently, through the analysis of our pedigrees, we have found several unaffected carriers of the c.286+2T>G and that aHUS penetrance was lower than 30%.

As a prototype of incomplete penetrance associated with c.286+2T>G RV, we investigated in depth a large pedigree (#646) from our cohort with nine carriers, of whom only one was affected by aHUS. The proband, as well as six unaffected family members, is heterozygous for the c.286+2T>G RV, and surprisingly, the healthy proband's father and aunt were even homozygous for this variant. Bathia et al. (26) described the incomplete penetrance of the c.286+2T>G RV in a family with three homozygous siblings, two of whom developed aHUS at 5 and 8 years of age but one of whom was still asymptomatic at age 10. The authors' hypothesis that the latter may develop HUS at a later age (26) is not supported by findings in our pedigree, showing two adults who are homozygous for this RV and are asymptomatic at 66 and 58 years of age at present.

Altogether, these data indicate that genetic deficiency of CD46 due to pathogenetic RVs is not enough to induce aHUS and that other genetic risk factors are likely important for disease manifestation.

This is consistent with the present and other published (12) findings that show that around 25% of aHUS patients with RVs in *CD46* carry a second or third RV in other complement genes. Specifically, in 23% of patients with the c.286+2T>G variant, we found RVs in other known disease-associated genes. However, this is not the case for the large pedigree #646 described here, since the proband did not carry other RVs or anti-FH antibodies.

In addition to complement gene RVs, common genetic modifiers, such as the *CFH–H3* and *CD46*_*GGAAC*_ haplotypes and the CFHR1^*^B allele, may influence susceptibility to aHUS (16, 32–34, 36). Indeed, the *CFH–H3* and *CD46*_*GGAAC*_ haplotypes and the homozygous CFHR1^*^B allele have been reported more frequently in aHUS patients than in controls. It is significant that our group and others have shown that in some aHUS families the disease manifested only in individuals who inherited a complement RV from one parent and the *CFH* and/or *CD46* risk haplotype from the other parent (32, 34, 42). However, the above findings are not universally true, since in other families aHUS penetrance was still incomplete in carriers of complement RVs and both *CFH–H3* and *CD46*_*GGAAC*_ risk haplotypes, suggesting that the synergic effect of these haplotypes may depend on the specific concurrent RV (12).

Consistently, here, we have found that the proband of the large pedigree with the 286+2T>G RV carries the *CFH–H3* risk haplotype, the homozygous *CD46*_*GGAAC*_ haplotype, and one copy of the CFHR1^*^B allele, but this combination does not explain penetrance, since the same risk factors are present in unaffected relatives who are carriers of the c.286+2T>G RV. The finding that in the other pedigrees with the c.286+2T>G RV the *CFH–H3* and *CD46*_*GGAAC*_ did not segregate with aHUS confirms that the above haplotypes do not have a significant effect on the risk of aHUS in *CD46* c.286+2T>G carriers.

c.286+2T>G is a splice variant and as such can affect mRNA processing and generate aberrant proteins with impaired structural and/or functional properties. However, different data on its effect have been reported. In one study, this RV was shown to cause an in-frame deletion generating a shorter CD46 protein that lacks part of SCR1 (24) and likely maintains a partial regulatory function. At variance, in another report, c.286+2T>G was shown to have a greater impact, resulting in the premature truncation of CD46 at SCR2 with the loss of all the other complement regulatory SCRs and of the transmembrane and intracellular domains (25). Thus, we wondered whether differences in the mRNA and protein products of the abnormal splicing between the proband and the unaffected carriers could explain incomplete aHUS penetrance in our pedigree. However, this was not the case, since we found that the proband and all healthy carrier relatives express mRNAs encoding both the shorter and truncated CD46 variants and that the latter was prominent in all carriers. The finding that the proband and the heterozygous c.286+2T>G carriers had approximately half-normal CD46 levels on blood cells and homozygous carriers had an almost total CD46 deficiency confirmed at the protein level that the truncated variant was the main product of the c.286+2T>G RV. Severe CD46 protein deficiency was also reported in a patient with aHUS and his healthy sister, both of whom were homozygous for another RV, *CD46* c.286+1T>G, affecting the same splice site (43). Together, these findings indicate that CD46 deficiency is not enough to induce the aHUS phenotype.

As multiple complement regulatory proteins, including the transmembrane decay-accelerating factor (DAF), protectin (CD59), and plasma factor H are expressed or bind to endothelial cell surfaces, the endothelium can regulate complement even when gene abnormalities cause impaired activity of one of them (44). This is supported by studies in blood outgrowth endothelial cells showing that the inhibition of CD46 with a specific antibody followed by exposure to normal human serum as a source of complement did not cause C3 deposition on cell surfaces (45).

In search of additional abnormalities in the proband of our pedigree that could predispose him to aHUS, we used an *ex vivo* assay of serum-induced complement activation on a cultured endothelial cell line (HMEC-1) [34], based on evidence that in aHUS the microvascular endothelium is the main target of complement dysregulation (2). In previous studies, the assay with ADP-activated HMEC-1 allowed us to specifically pick up genetic complement abnormalities that affect circulating regulators both in aHUS patients and in their unaffected relatives (37).

The finding here that serum from the proband, and also sera from the mother and a maternal uncle, caused extensive C3 and C5b-9 deposits on activated HMEC-1 indicates that the proband inherited from his mother a genetic abnormality that causes complement activation on endothelial cells.

At variance, the unaffected paternal relatives carrying the c.286+2T>G *CD46* RV had normal serum-induced complement deposition on endothelial cells, which is an expected finding because CD46 is a surface complement regulatory protein and the endothelial cell line used in the assay expresses normal CD46. Thus, the proband has two main genetic predisposing factors, the c.286+2T>G *CD46* RV inherited from his father and a defect of maternal lineage that remains unknown and which, together with the *CFH–H3* and *CD46*_*GGAAC*_ risk haplotypes, have synergized and induced aHUS. The finding that both the paternal relatives carrying the c.286+2T>G *CD46* variant and the maternal relatives with an abnormal complement deposition test are unaffected indicates that either genetic defect alone were not enough to induce aHUS.

Whole exome sequencing studies in this pedigree are required to identify the genetic defect inherited from the patient's mother, using segregation with the phenotype “elevated complement deposits” as criterion for selection of candidate variants.

Terminal complement activation associated with aHUS has been shown to activate platelets and neutrophils and to induce the formation of neutrophil-platelet aggregates (46, 47). Further *ex vivo* studies with platelets and blood leukocytes from the proband and his relatives could also contribute to clarify the complex pathogenetic mechanisms leading to aHUS in this pedigree.

aHUS patients with *CD46* RVs usually have a good prognosis. Although recurrences are frequent, spontaneous remissions are common and the long-term outcome appears to be good, with about 90% of patients maintaining normal renal function. The proband described here, however, had a severe disease course and developed ESRF, despite intensive plasma-exchange therapy. This phenotype closely resembles that of patients with combined RVs in *CD46* and other complement genes (14), thus supporting the hypothesis that the proband inherited an additional genetic defect from his mother. The observation that the proband did not experience HUS recurrence in the kidney graft and exhibits good a graft function at the 5-year follow-up after transplantation can be explained by the transplant correcting the *CD46* genetic defect, since endothelial cells within the kidney allograft express normal CD46. This finding also supports our interpretation that both the paternal CD46 variant and the unidentified maternal defect synergized to induce aHUS in this pedigree.

In conclusion, we demonstrate that *CD46* c.286+2T>G is a variant with a severe functional effect and also confirm that CD46 deficiency alone is not sufficient to develop aHUS, even though it is a strong risk factor.

The results from this study are consistent with earlier reports in the literature (33) that have documented the complexity of genetic abnormalities associated with aHUS, which range from highly penetrant RVs and genomic rearrangements that affect *CFH* to RVs that cause aHUS only in the presence of other RVs and/or risk haplotypes. Combining genetic studies with the *ex vivo* test of complement activation on endothelium may contribute to explaining the determinants of penetrance in this complex disease.

## Data Availability Statement

The raw data supporting the conclusions of this article will be made available by the authors, without undue reservation, to any qualified researcher.

## Ethics Statement

The studies involving human participants were reviewed and approved by Ethics Committee of the Azienda Sanitaria Locale, Bergamo, Italy. Written informed consent to participate in this study was provided by the participants' legal guardian/next of kin.

## Author Contributions

RP, MN, and GR designed research, interpreted data, and wrote the paper. RP, PI, MT, SG, EV, MA, CM, MG, and PC performed the research and analyzed the data. EB provided detailed clinical information of patients. AB analyzed the data and critically revised the manuscript. All authors contributed to the article and approved the submitted version.

## Conflict of Interest

MN has received honoraria from Alexion Pharmaceuticals for giving lectures and for participating in advisory boards. MN has received research grants from Omeros, Alnylam, and ChemoCentryx. GR has consultancy agreements with AbbVie, Alexion Pharmaceuticals, Bayer Healthcare, Reata Pharmaceuticals, Novartis Pharma, AstraZeneca, Otsuka Pharmaceutical Europe, and Concert Pharmaceuticals; for which no personal remuneration was accepted, and compensation is paid to his institution for research and educational activities. The remaining authors declare that the research was conducted in the absence of any commercial or financial relationships that could be construed as a potential conflict of interest.

## Supplementary Material

Supplementary Figure 1 Gating strategy used to evaluate CD46 surface expression on lymphocytes by flow cytometry. Fresh or thawed PBMCs were labeled with anti-human CD3 antibody and with antibody anti-human CD46 FITC recognizing SCR1 epitope **(A)** or with antibody anti-human CD46 FITC recognizing SCR4 epitope **(B)**.
