# Supplementary figures and images for: Molecular Studies and an ex vivo Complement Assay on Endothelium Highlight the Genetic Complexity of Atypical Hemolytic Uremic Syndrome: The Case of a Pedigree With a Null CD46 Variant
Source: Front Med (Lausanne). 2020 Nov 3;7:579418. doi: 10.3389/fmed.2020.579418 (PMC7670076; doi:10.3389/fmed.2020.579418)

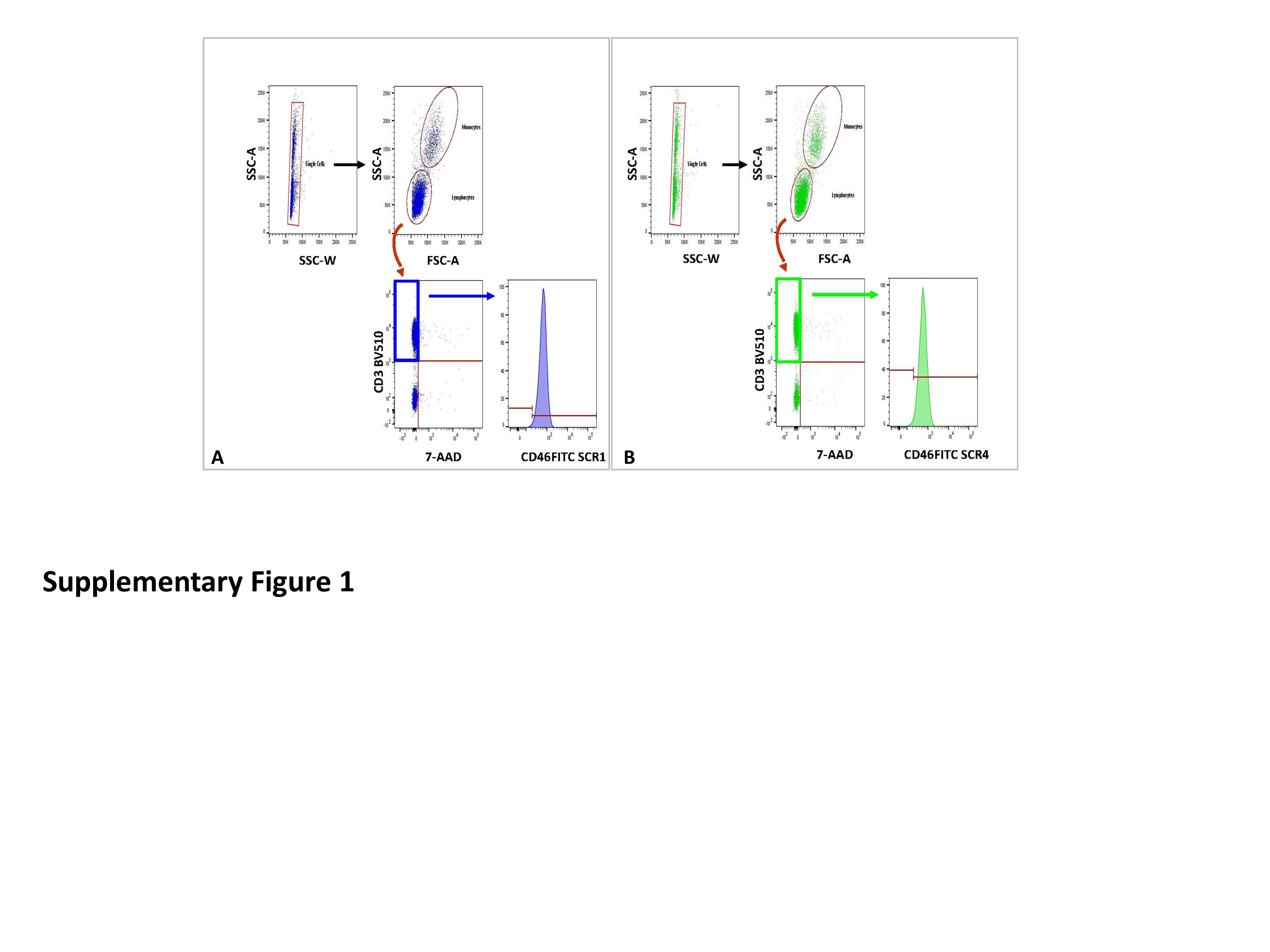

Supplement: Supplementary Figure 1 — Gating strategy used to evaluate CD46 surface expression on lymphocytes by flow cytometry. Fresh or thawed PBMCs were labeled with anti-human CD3 antibody and with antibody anti-human CD46 FITC recognizing SCR1 epitope (A) or with antibody anti-human CD46 FITC recognizing SCR4 epitope (B). [file Image_1.TIF]
